# A Mixed-Methods Evaluation of a Project ECHO Program for the Evidence-Based Management of Sickle Cell Disease

**DOI:** 10.3390/ijerph21050530

**Published:** 2024-04-25

**Authors:** Cami Mosley, Christina Bennett Farrell, Charles T. Quinn, Lisa Marie Shook

**Affiliations:** 1 Cincinnati Comprehensive Sickle Cell Center, Division of Hematology, Cincinnati Children’s Hospital Medical Center, Cincinnati, OH 45229, USA; 2 Department of Pediatrics, College of Medicine, University of Cincinnati, Cincinnati, OH 45229, USA

**Keywords:** sickle cell disease, healthcare provider, provider education, continuing education, evidence-based management, telementoring

## Abstract

Sickle cell disease (SCD) is a group of chronic, genetic disorders of the red blood cells with significant gaps in access to evidence-based clinical care. Sickle Treatment and Outcomes Research in the Midwest (STORM), a provider network, utilized Project ECHO (Extension for Community Health Outcomes), a telementoring model, to deliver evidence-based education about SCD management. The purpose of this mixed-methods study is to evaluate the utility of Project ECHO as an educational strategy for healthcare providers treating children and adults with SCD. Annual evaluations were administered to STORM TeleECHO participants from 2016 to 2021. Survey data showed a statistically significant change in self-reported provider confidence in the ability to provide care for adult patients with SCD; identify suitable candidates for disease-modifying therapies; and confidence to prescribe disease-modifying therapies. Participants who attended at least 10 sessions were invited to participate in a semi-structured interview. Qualitative data were analyzed using thematic analysis and several themes emerged about the benefits, including (1) increased confidence, (2) integrated best-practice care, (3) connection to provider network and access to experts, (4) high-quality educational presentations and (5) opportunities for collaboration and a sense of community. This suggests that Project ECHO is accessible and leads to increased confidence in providers caring for individuals with SCD. Overall, participant knowledge gains successfully demonstrated the utility of Project ECHO as an educational resource for providers.

## 1. Introduction

In the United States, there are an estimated 100,000 individuals living with sickle cell disease (SCD), with approximately 15,000 of those individuals in the Midwestern region [1]. SCD is a group of chronic, genetic disorders of the red blood cells that are usually diagnosed at birth through newborn screening [2]. Twenty-year historical data from state newborn screening programs have shown its detection in approximately 1 in 2000 US newborns (with increased detection of about 1 in 365 Black newborns) [3]. SCD is characterized by chronic organ complications, pain crises, and comorbidities for healthcare providers to manage throughout childhood and as patients transition to adult care [4,5]. Complex chronic disorders, such as SCD, can be challenging for healthcare providers to manage clinically, especially if they see few patients with SCD in their practice and as many more patients live well into adulthood [6]. Studies have found that healthcare providers often self-report a limited knowledge of evidence-based practices and low confidence in treating patients with SCD, which often leads to a lack of access to high-quality care [7].

A strategy to increase provider knowledge and confidence in treating SCD across the lifespan is to implement evidence-based best practices and guidelines for clinical care. One example of clinical guidelines development is the efforts of the National Heart, Lung and Blood Institute (NHLBI) of the National Institutes of Health, which convened a panel of subject matter experts to develop evidence-based guidelines for the management of SCD in 2014. These guidelines included recommendations for SCD-specific screenings and health maintenance, the prescribing and monitoring of treatment such as hydroxyurea, and managing acute care [8,9]. From 2019 to 2021, the American Society of Hematology released several additional clinical guidelines for the management of SCD, including cardiopulmonary and kidney disease [10], acute and chronic pain [11], cerebrovascular disease in children and adults [12], transfusion support [13], and stem cell transplantation [14]. While crafting evidence-based guidelines is a first step to higher-quality care, research has shown that it can take up to 17 years to translate 14% of published evidence into practice [15].

Moreover, until recently, treatment options for SCD have been limited as hydroxyurea was the only FDA-approved disease-modifying therapy for SCD since 1998. However, over the past few years, the FDA has approved several other treatments for SCD, including L-glutamine (2017), crizanlizumab, voxelotor (2019), and most recently, gene therapies (Exa-cel and Lovo-cel, 2023) [16]. Studies over the past decade have shown that hydroxyurea, which is a daily oral medication, has consistently been underprescribed by healthcare providers [17]. Despite studies that have demonstrated hydroxyurea can significantly reduce hospitalizations, blood transfusions, pain, and other complications and also significantly increase survival for both pediatric and adult patients, it remains underutilized. Studies have shown that as few as 20–30% of eligible adult patients are prescribed this medication in the US [18,19]. Moreover, studies have shown that one of the key drivers for underprescribing hydroxyurea is limited provider knowledge and low comfort level with identifying eligible patients, prescribing the medication, and monitoring the dosing of hydroxyurea. 

Sickle Treatment and Outcomes Research in the Midwest (STORM) is a regional sickle cell learning network of pediatric and adult healthcare providers that aims to improve outcomes for children and adults with SCD. STORM has been funded since 2014 as part of the HRSA Sickle Cell Disease Treatment Demonstration Project (SCDTDP) grant. STORM spans ten clinical sites in eight states in the Midwest (Indiana, Illinois, Michigan, Minnesota, North Dakota, Ohio, South Dakota, and Wisconsin). A key aim of the project is to increase provider knowledge about evidence-based management of SCD and increase the number of patients receiving high-quality care across the lifespan. In 2016, the STORM network launched a regional virtual telementoring program utilizing the Project ECHO (Extension for Community Healthcare Outcomes) framework [20].

Project ECHO is an innovative telementoring approach that can link multidisciplinary specialists with other healthcare providers regularly via videoconference to engage in didactic presentations and case-based learning [21]. ECHO has successfully been implemented across multiple diseases and conditions to supplement knowledge and enhance the self-efficacy of healthcare providers. Moreover, the ECHO model creates a community of practice where knowledge is shared freely and focuses on an “all-teach, all-learn” culture. The ECHO model was originally designed to link sub-specialists and primary care providers to deliver educational content [21,22,23,24,25,26]; however, the participant uptake for the STORM ECHO included primarily pediatric and adult hematologists, multidisciplinary sickle cell clinic team members, and very few primary care providers (PCPs), despite targeted recruitment efforts to primary care providers. 

One of the key principles of the Project ECHO model is to utilize low-cost Zoom^®^ technology to deliver educational content in a way that is easily accessible [21,22,23,24,25,26]. The Project ECHO format includes live monthly sessions that are 60 min in duration, including a 30–45 min didactic presentation, followed by a case presentation with facilitated discussion. Pediatric and adult cases that are de-identified and HIPAA-compliant can be presented by participants using a template that includes relevant medical history, including laboratory results, current treatment plan, and psychosocial history. The goal of case discussions is to provide feedback to the presenter about complex cases from psychosocial and medical perspectives. During the facilitated case discussion, all participants may ask questions and are encouraged to provide treatment recommendations. Case presentations are a key strategy to build a “community of practice” among providers, and to serve as an essential clinical resource for healthcare providers. STORM TeleECHO is an open-session, ongoing educational program, which allows participants the autonomy to select relevant session topics and to attend when their schedule allows.

The STORM TeleECHO curriculum is grounded in evidence-based treatment and management including preventative care, management of complications, disease-modifying therapy treatments, and psychosocial issues to improve provider knowledge and comfort in caring for patients with SCD, leading to improved clinical outcomes. Didactic topics presented by subject-matter experts over the evaluation period included overarching themes of pediatric-focused medical management; adult-focused medical management; psychosocial management and support across the lifespan; health equity; and COVID-19 and SCD (Table 1). Educational presentations are recorded and archived on the STORM SharePoint site. Survey tools are used to measure the impact of STORM TeleECHO sessions on provider knowledge, comfort level, and practice behavior changes. Continuing education credits are provided for attendees, including continuing medical education (CME), American Board of Pediatrics, American Board of Internal Medicine Maintenance of Certification Part II (MOC), and nursing credits.

This mixed-methods study evaluates the utility of Project ECHO as an educational strategy for healthcare providers treating children and adults with SCD over a five-year period. The study also evaluates self-reported impact on provider knowledge and comfort with managing SCD.

## 2. Materials and Methods

### 2.1. Design and Setting

For the quantitative data collection, six-month interval evaluations were sent via a Survey Monkey © web-based survey to assess changes in practice, clinical expertise, and knowledge of evidence-based guidelines for SCD. Multidisciplinary healthcare providers who attended at least two STORM ECHO monthly sessions during a calendar year received the survey via email. 

For the qualitative data collection, the study used semi-structured interviews with a framework that was informed by the results of yearly STORM program evaluation surveys conducted between 2016 and 2021. Study participants were sourced via convenience sampling of any healthcare provider that participated in at least 10 of 67 possible STORM TeleECHO sessions held from March 2016 to March 2021, as well as participants who indicated on a program evaluation survey a willingness to be contacted for evaluation interviews. Study participants were recruited from STORM TeleECHO via an invitational study email. Interviews were conducted online utilizing the Zoom© web conference platform from May 2021 to November 2021. All study participants were incentivized with USD 25 for their time.

### 2.2. Ethical Considerations

The study was approved by the Cincinnati Children’s Hospital Medical Center IRB. Permission was obtained to conduct the research (survey and semi-structured interviews). Participants in the semi-structured interviews gave electronic informed consent, including voluntary participation and the right to withdraw from the study at any time.

### 2.3. Data Collection

For the quantitative data collection, participants completed a self-reported demographic and registration survey in Survey Monkey® prior to joining their first ECHO session. All participants received an annual survey about the educational program via email to complete in Survey Monkey®. The online STORM ECHO registration survey, also completed in Survey Monkey®, asked participants to self-report across a number of domains using a confidence scale: (1 = Not at all confident to 4 = Very confident). Participants reported confidence with (1) providing care for children with SCD; (2) providing care for adults with SCD; (3) managing acute pain in children with SCD; (4) managing acute pain in adults with SCD; (5) managing chronic pain in children with SCD; (6) managing chronic pain in adults with SCD; (7) identifying eligible candidates for disease-modifying therapies, such as hydroxyurea; (8) prescribing and monitoring dosing of hydroxyurea and other disease-modifying therapies; and (9) serving as a healthcare provider for SCD patients. Participants who attended at least two sessions annually were invited to complete follow-up surveys at multiple time points (e.g., 6 months, 12 months, 18 months, 24 months, and 36 months) to self-report their confidence after attending ECHO sessions.

For the qualitative data collection, a clinical research coordinator trained in focus group facilitation and qualitative research conducted the semi-structured interviews online using the Zoom® web-based video conferencing platform. Interviews lasted approximately 1-h and were digitally recorded for professional transcription. The semi-structured interviews focused on the providers’ experience participating in the STORM TeleECHO program as well as a discussion about the preferred methods and content of continuing education regarding SCD for healthcare providers.

### 2.4. Data Analysis

A Wilcoxon signed-rank test [27], a non-parametric test, was used to test for differences between paired (before–after) measurements for the quantitative evaluations.

For the qualitative data, an inductive analysis based on a content analysis [28] was conducted. Inductive analysis included three phases: preparation, organizing, and reporting the data. In phase 1, interview transcripts were coded into main themes. In phase 2, data was categorized into main categories along with overall themes. To ensure quality assurance, an additional coder independently analyzed the transcripts and coding. Finally, a study team meeting was held to debrief and discuss emerging findings. In the last analysis phase, coded text was reviewed for final themes.

## 3. Results

There were 230 unique participants that attended STORM ECHO during the 5-year period. A total of 34 participants were included in the survey data analysis for eligibility criteria of (1) completing the survey at two different time points and (2) attending at least two ECHO clinics over a period of 5 years. The median time (124 days–2182 days) between time points was 1115 days, or approximately 3 years. The mean number of sessions attended by survey participants was 26 sessions out of a possible 68 sessions. 

Survey participants were a sampling of multidisciplinary providers, including physicians, nurse practitioners, registered nurses, newborn screening coordinators, etc. (Table 2). Half of the participants (*n* = 17, 50%) reported being under 50 years old and had less than 20 years of experience treating patients with SCD. Over one-third of providers were people of color (i.e., Asian or Black/African American; *n* = 13, 38.2%). A Wilcoxon signed-rank test showed that ECHO clinic participation elicited a statistically significant increase in confidence in (a) providing care for adult patients with SCD (Z = −2.152, *p* = 0.031); (b) ability to identify suitable candidates for disease-modifying therapies, such as hydroxyurea (Z = −3.034, *p* = 0.002); and (c) prescribing disease-modifying therapies (Z = −3.116, *p* = 0.002).

Overall, program evaluations showed that by participating in STORM TeleECHO, 92% of participants learned best practice care for SCD, 94% learned with guidance from specialists in SCD management, and 81% reported developing clinical expertise. Moreover, 90% of participants reported that their patients also benefit from participation in STORM TeleECHO by applying best practice care learned at ECHO.

Six semi-structured interviews were conducted with various multidisciplinary providers that attended a range of 12 sessions to 62 sessions out of a possible 68 sessions over the five-year period (Table 3). The majority of interview participants were pediatric hematologists (*n* = 3), and there was also a pediatric primary care provider (*n* = 1). The six participants included five clinicians, including three pediatric hematologists, a nurse practitioner, and a pediatric primary care provider. Four of the clinicians provided estimates about the number of SCD patients they serve, ranging from 2 to 200 patients (average = 96 patients). The average number of years of experience was 23 years, ranging from 12 to 36 years. There was not a correlation between the number of years of experience treating individuals with SCD and the number of sessions that providers attended.

The average number of years of experience amongst the five clinicians was 23 years, ranging from 12 to 36 years. There was not a correlation between the number of years of experience treating individuals with SCD and the number of sessions that providers attended.

Several main themes from the qualitative interviews emerged, including increased healthcare provider confidence, integrated best-practice care, connection to a network of providers, and the quality of session content.

### 3.1. Increased Confidence

Participants described how the ECHO sessions, specifically the case presentations, helped build confidence in clinical decision-making in the management of SCD.

*“…it’s helped me develop more confidence… When they provide advice, I’m like, “Okay, good. That’s what I was thinking too.” Then it gives me more confidence that I actually do know what I’m doing most of the time.”*

*“…I definitely feel more well-rounded, more confident absolutely…”*

### 3.2. Integrated Best-Practice Care

Participants described how attending ECHO sessions increased their understanding of best practices and evidence-based guidelines for SCD. As a result, they self-reported changing clinical practices, which will lead to better, high-quality care for patients.

*“…it just enhances my understanding of multiple approaches and again, best practices, standards of care… I think that there are some subtle ways that it does. I think it just challenges you to be the best version and make sure that all of your protocols and standards are up to date.”*

*“[My clinical practice] is more aligned with best practices and it’s really grown who I am and how I think critically, which I think also has changed the way that I practice. I don’t just see a patient and continue the plan as it stands. I’m really thinking about is this currently what best practices would consider and I’m thinking about things that I might not have otherwise thought about because of my participation in STORM.”*

### 3.3. Connection to Provider Network

Participants described the community of practice created by STORM ECHO and the benefit of having a safe space to discuss complicated cases in order to receive clinical feedback from other providers and subject-matter experts and to have other experts validate their own clinical decision-making. Providers also described feeling disconnected and isolated from others treating patients with SCD and appreciated the opportunity to establish virtual relationships with other SCD providers through STORM ECHO.

*“I think we’ve developed a nice network. I think that we trust each other, and we trust each other’s advice. I think that everyone is given the opportunity to contribute, which I appreciate. And people’s opinions and views are respected.”*

*“I think it makes it easier that if you do have a complicated case in between TeleECHO sessions to send that email and you [have] a network of people that are willing to help you.”*

*“So, not only is it the broadening of my own network and name recognition, role recognition, and connecting, but it’s also—it allows me in my state to see how someone in another state may do something differently so that we don’t get siloed into the belief that how we do it is the only way to do it.”*

*“…I feel that this is the comradery in that we are talking about the same problems, we are asking the same questions, we are trying to learn, we are trying to study the things that we commonly have questions for…”*

*“I think having that community to tap into to run a case by another expert in the field that’s not local, it’s like a second opinion, but in a way not because it’s not inconveniencing the patients and it’s really giving them the best care possible.”*

### 3.4. Presentation Quality

Participants described the value of the STORM ECHO session didactic presentations, including presenters being nationally recognized subject matter experts, and described the utility of the evidence-based content presented in a practical manner for implementation into practice in their own clinical settings. Participants also found value in the framework of Project ECHO sessions, which are balanced between both the didactic presentation and case presentation.

*“…a lot of it balanced between the cases… and the actual didactic content, which I found to be enough that I took notes from it and kept the notes. It was very helpful information.”*

*“I think the didactic sessions are always quite good… And the presenters are always top-notch, excellent. So, that’s a big strength. I think the cases are a big strength, too, considering a variety of cases…”*

*“…my favorite thing is that you have different experts across the country presenting and I feel like the anecdotal experience is tangible because people utilize patient cases that are very relatable. The other piece that’s really, really important to me data-driven practice and I feel like that definitely comes through in the STORM presentations, that people are being data-driven…”*

*“I think the fact that the didactic presentations are incredibly practical, they’re short, usually 20 or 30 min, so they’re very distilled into what you really need to know, and I think that adds value. The ability to stop and ask questions about halfway through before you do the case, I think, is very valuable.”*

*“I think the way that ECHO is packaged is bite-sized and it works for a busy clinician, and I think that’s a great idea.”*

*“I think the predictability, the practicality, and the boundaries of it make it a very pleasant way of learning…”*

## 4. Discussion

This mixed-methods evaluation suggests that Project ECHO telementoring is a successful, sustainable program that is acceptable to healthcare providers as an innovative strategy to provide continuing education and training about the evidence-based management of SCD. Through qualitative and quantitative evaluation methods, STORM ECHO participants were able to provide evidence of the acceptability and overall positive impact of the educational program on increasing knowledge and comfort level while managing the treatment of SCD. Participants self-reported changes in practice and behavior, specifically around increased confidence in identifying patients eligible for hydroxyurea and increased confidence in prescribing and monitoring the dosage of this disease-modifying therapy. Participants noted the value of building a “community of practice” for pediatric and adult multidisciplinary healthcare providers managing SCD.

There are several limitations to this study. One notable limitation is that while the providers who were interviewed had attended numerous STORM ECHO sessions, this study represents a small sample size of healthcare provider qualitative interviews. Additional studies to understand the impact on patient care and provider clinical decision-making are needed. For example, in some chronic condition Project ECHOs, there can be hundreds of participants per session, with the majority of participants representing primary care. However, the effectiveness and success of ECHO sessions for rare disorders may need different measurements for impact other than the number of participants attending each session. Collecting low-burden data about how many patient lives have been reached by the STORM TeleECHO would be helpful as a next step. 

Further, while STORM ECHO participants self-reported their intentions to apply new knowledge and evidence-based practices, including an increase in prescribing hydroxyurea and other disease-modifying therapies for eligible patients, a data collection method is needed to determine whether these self-reported findings actually translate into clinical practice and improved patient outcomes for children and adults with SCD. Moreover, the overall response rate during this time period for the quantitative and qualitative study was limited. One reason for this limited response rate could be the competing demands of the COVID-19 pandemic, which occurred during the study period. 

## 5. Conclusions

This study demonstrates the utility of Project ECHO as a successful educational strategy for sub-specialists, which is an adaptation from the original Project ECHO framework that was designed to focus educational efforts on primary care providers, to learn about the management and co-management of disorders with sub-specialists [21]. Targeted efforts to engage primary care providers in the STORM ECHO sessions did not result in a high uptake. However, the participant reach of STORM ECHO has continued to consistently grow, with additional pediatric and adult hematologists and multidisciplinary sickle cell clinic team members (i.e., psychologists, pharmacists, care managers, social workers, etc.) joining the educational program. STORM ECHO will continue to use formative evaluation methods to inform the didactic curriculum and ensure this educational framework continues to meet learner needs and increase provider knowledge and self-confidence about the management of SCD. This educational strategy can be useful to continue to disseminate evidence-based guidelines and treatment recommendations for new disease-modifying therapies for SCD, including the FDA approval of additional disease-modifying therapies over the past few years [29] and the recent FDA approval of gene therapy.

## Figures and Tables

**Table 1:** STORM TeleECHO Didactic Presentation Topics (March 2016–March 2021).

Year	Topic	
2016	Pediatric Complications Newborn Screening Follow-up Psychosocial Issues Adult Complications Hydroxyurea	Abdominal Complaints Renal Complications of SCD Pain in SCD Retinopathy Transfusions
2017	Home Pain Management Plans Acute Chest Syndrome Avascular Necrosis Pulmonary Hypertension Hydroxyurea 2.0 Pregnancy and Reproductive Disorders	Priapism Endari—a New FDA Approved Medication Reproductive Issues and Contraception Cerebrovascular Phenotypes CNS Complications
2018	Pain Management and Prescribing Titrate Opioids Pediatric Medication Adherence Cerebrovascular Phenotypes Transition from Pediatric to Adult Care Racial Health Equity: Implications for SCD	Health-related Quality of Life in SCD Prevention of Invasive Bacterial Infections with SCD Jehovah’s Witness and Blood Products Cognition and Education of Students with SCD Bone Marrow Transplant
2019	Asthma in the SCD population SCD and Dental Issues Sickle Cell Trait and Renal Medullary Carcinoma Case Presentation Spotlight and Updates	Living Fully with SCD Primary and Secondary Pediatric Co-management Iron in SCD SCD and Exercise Cannabinoids and SCD
2020	* COVID-19 Open Forum Part 1 * COVID-19 Open Forum Part 2 * Telemedicine Best Practices * Sickle Cell Disease Association of America’s COVID-19 Patient and Provider Advisories * The SCD COVID-19 Surveillance Registry Sickle Cell Data Collection (SCDC): The Intersection of Surveillance and Clinical Care * Blood Safety and Transfusion Practices * Serology for SARS-CoV-2 * Lessons from the Field: Telemedicine Approach to Geriatric SCD * Mental Health and Coping During COVID-19 * COVID-19-associated Multi-system Inflammatory Syndrome in Children (MIS-C)	* Are Children with SCD Immune to COVID-19? ASH Guidelines for Transfusion COVID-19 is not the only pandemic facing our SCD families: A forum to discuss racism—the Minneapolis experience. New ASH Guidelines for Cerebrovascular Disease * Back to School Planning for Students with SCD New ASH Guidelines for Cardio-pulmonary and Kidney Disease * An Update on COVID 19 and SCD Patient Outcomes Vitamin D and SCD New ASH Clinical Guidelines for Pain Perceived Racial Bias and Health Related Stigma in Pediatric SCD
2021	* Sickle Cell Disease Association of America—Recommendation for COVID Vaccination Lead Poisoning and SCD	* COVID-19 Vaccine Update

* Denotes COVID-19 and Sickle Cell Disease session.

**Table 2:** Demographic characteristics of survey participants.

Demographics	N	%
Training		
Doctor (MD, DO)	18	52.9%
Nurse Practitioner	4	11.8%
Physician’s Assistant	2	5.9%
Registered Nurse	3	8.8%
Other (MPH, BSN, Program Coordinator, Research Coordinator, Newborn Screening Coordinator, etc.)	6	17.6%
Position		
Specialist/sub-specialist in hematology	2	5.9%
Specialist/sub-specialist in pediatric hematology	15	44.1%
Specialist/sub-specialist in adult hematology	1	2.9%
Primary care provider who sees children, youth, and adults	1	2.9%
Primary care provider who exclusively sees children and youth	3	8.8%
Primary care provider who exclusively sees adults	1	2.9%
Other (Research Coordinator, Program Coordinator, Consultant, Pastor, etc.)	10	29.4%

**Table 3:** Demographic characteristics of healthcare provider interview participants.

Number of Sessions Attended	Training	Position	Years of Experience	Age	Race
18	Doctor (MD, DO)	Primary care provider who exclusively sees children and youth	41	60+	White
49	Doctor (MD, DO)	Specialist/sub-specialist in pediatric hematology	51	60+	Asian
46	Doctor (MD, DO)	Specialist/sub-specialist in pediatric hematology	18	40–49	White
14	Community Health Worker	Community Health Worker	17	40–49	Black or African American
12	Nurse Practitioner	Specialist/sub-specialist in pediatric hematology	*	*	*
62	Doctor (MD, DO)	Specialist/sub-specialist in pediatric hematology	36	60+	White

* Information not reported.

## Data Availability

The data presented in this study are available on request from the corresponding author.
